# A curated and standardized adverse drug event resource to accelerate drug safety research

**DOI:** 10.1038/sdata.2016.26

**Published:** 2016-05-10

**Authors:** Juan M. Banda, Lee Evans, Rami S. Vanguri, Nicholas P. Tatonetti, Patrick B. Ryan, Nigam H. Shah

**Affiliations:** 1 Center for Biomedical Informatics Research, Stanford University, Stanford, California 94305, USA; 2 LTS Computing LLC, West Chester, Pennsylvania 19380, USA; 3 Department of Biomedical Informatics, Columbia University, New York, New York 10032, USA; 4 Janssen Research & Development, LLC, Titusville, New Jersey 08869, USA

**Keywords:** Translational research, Outcomes research, Adverse effects, Drug safety

## Abstract

Identification of adverse drug reactions (ADRs) during the post-marketing phase is one of the most important goals of drug safety surveillance. Spontaneous reporting systems (SRS) data, which are the mainstay of traditional drug safety surveillance, are used for hypothesis generation and to validate the newer approaches. The publicly available US Food and Drug Administration (FDA) Adverse Event Reporting System (FAERS) data requires substantial curation before they can be used appropriately, and applying different strategies for data cleaning and normalization can have material impact on analysis results. We provide a curated and standardized version of FAERS removing duplicate case records, applying standardized vocabularies with drug names mapped to RxNorm concepts and outcomes mapped to SNOMED-CT concepts, and pre-computed summary statistics about drug-outcome relationships for general consumption. This publicly available resource, along with the source code, will accelerate drug safety research by reducing the amount of time spent performing data management on the source FAERS reports, improving the quality of the underlying data, and enabling standardized analyses using common vocabularies.

## Background and Summary

Adverse drug events (ADEs) are defined as injuries resulting from medication use, from which adverse drug reactions (ADRs) are ADEs that occur due to the pharmacologic properties of the drugs involved. According to studies^1^, the annual cost of drug-related morbidity and mortality was estimated to be around $170 billion and rising in 2000. For 2012, the most recent year for which data are available, the agency for healthcare research and quality estimated that more than 1.9 million emergency room visits in the United States are related to ADRs^2^.

Pharmacovigilance refers to the science relating to the detection, assessment, understanding and prevention of adverse effects or any other drug-related problem. The Council for International Organizations of Medical Sciences (CIOMS) defines a safety signal as ‘information that arises from one or multiple sources (including observations or experiments), which suggests a new, potentially causal association, or a new aspect of a known association between an intervention [e.g., administration of a medicine] and an event or set of related events, either adverse or beneficial, that is judged to be of sufficient likelihood to justify verificatory action.’ Efficient and reliable identification and evaluation of ‘safety signals’ requires access to evidence from disparate sources, including electronic health records^3–6^, spontaneous reporting systems (SRS)^7–9^, social media^10–14^, literature mining^15–18^, web search queries via search engine logs^19–22^, and biological and chemical knowledge bases^23–25^. The belief is that each data source provides a unique vantage point in understanding a drug’s safety profile. Spontaneous adverse event reporting data have served as the cornerstone for signal detection activities, and have proven to be a useful source of evidence in the safety evaluation process. As such, safety scientists have come to rely on SRS a primary means of monitoring the safety of medical products. Increasingly, researchers who are exploring new analytical approaches and novel data sources to support pharmacovigilance have looked to SRS data as a benchmark and means of methodological evaluation. The widespread use of the FAERS data by drug safety researchers highlights the importance of the resource presented in this work.

Although a free and publicly available resource, the FDA FAERS data still presents multiple hurdles in consolidating all relevant data, normalizing different term usage, de-duplicating records, and mapping to either RxNorm (for drugs) or any other controlled terminology (for adverse events). Some additional data cleaning and imputation of missing values is also needed to take full advantage of the dataset. Research groups may perform some (or all) of these tasks when using the data for their studies, but this process represents a major time-sink; in addition, such repeated one-off efforts create the potential risk of some of the steps not being done properly further delaying progress or producing unreliable and irreproducible results. Over the years many private companies have curated and standardized this publicly available data into private resources charging a considerable fee for their efforts^26–30^. We are offering this resource in cleaned up form for free public download along with the code necessary to redo the cleanup steps as more data is made available in the future.

With the development of large community efforts such as the Observational Health Data Sciences and Informatics (OHDSI) initiative^31^, avoiding the need to re-process, clean and standardize the FDA’s FAERS data will reduce the amount of wasted effort put into these tasks and allow researchers to focus on learning insights from the data. In particular, those researchers who are interested in using statistics derived from FAERS, but don’t have the capacity to generate those statistics themselves, would find a common, standardized representation of evidence from FAERS more useful to apply into their activities. A resource that contains both standardized reports and pre-computed statistics from those standardized reports could enable research across an array of different domains. We name our resource as AEOLUS, which stands for **A**dverse **E**vent **O**pen **L**earning through **U**niversal **S**tandardization.

The broader community will greatly benefit as the resource will be available to any independent researcher, reducing the number of independent curations of the dataset and increasing the reproducibility of research findings. Finally, such open data sharing embodies the intent of efforts such as www.healthdata.gov, which aim to enhance use of publically available datasets from the US government.

## Methods

**Figure 1:**
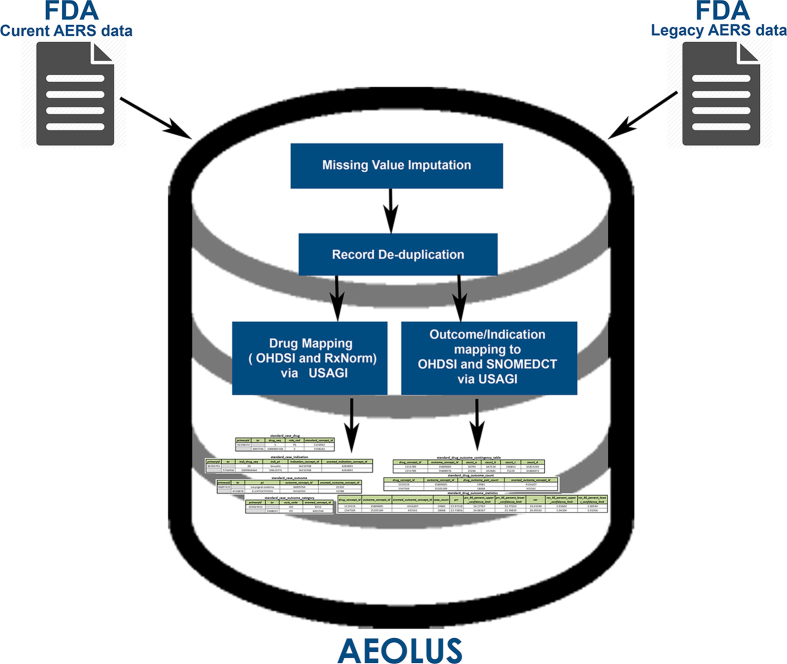
AEOLUS Integration and generation process.

The FDA’s Adverse Reporting System data is publicly available as a quarterly downloads on the FDA’s website (http://goo.gl/9Lcc65), two formats: Extensible Markup Language (XML) and Comma Separated values files (CSV). For researchers wanting to use all available data the FDA provides legacy data, which we will call LAERS, which covers from January 2004 to August 27, 2012. This legacy data introduces the first challenge as it is on a slighter different data format as the most current data, which we will call FAERS, and covers from September 2012 until June 2015 (at the time of writing). The data found in LAERS/FAERS comprises adverse events and medication errors reported by healthcare professionals (pharmacists, nurses, physicians) and consumers (patients, lawyers, family members) on a voluntary basis in the United States. It is important to note that if a manufacturer receives an adverse event report from consumers or healthcare professionals, they are required, by regulations, to send the report to FDA. All of these reports are then compiled by the FDA into one resource, leading to the challenge of de-duplicating some of the reports. Figure 1 presents and outline of the steps taken to build our dataset.

### FAERS/LAERS source data

Once downloaded and extracted, each of the quarterly FAERS/LAERS data files are divided in seven individual tables as described in Table 1. Each table can be loaded onto a database or manipulated directly.

The main differences between LAERS and FAERS data lies in the renaming of the key fields: *isr* and *case* to *primaryid* and *caseid* respectively. In our resource when joining both sets of data we keep both names present to allow researchers to trace the reports back to their original data source. There are extra fields added between the different sets of data, but as they don’t play a role on our data processing, we refer the reader to the documentation included with the FDA source files for details.

We used the DEMOyyQq tables in our missing value imputation and case de-duplication steps. We provide enhanced and integrated versions of the DRUGyyQq, INDIyyQq tables that include mappings to OHDSI standard concept identifiers via RxNorm Concept Unique Identifiers (CUIs) and SNOMED-CT identifiers respectively. From the original DRUGyyQq we mapped, when possible, the textual drug names to OHDSI standard concept identifiers via five different steps as indicated in the Drug Mappings section. A similar process using MedDRA codes, is outlined in the Indication and Reaction mappings section for the INDIyyQq mapping of the drug indications. The process behind the merging and data mappings is outlined in the remaining subsections.

### Data merging

As the first step in the data curation process, both LAERS and FAERS drug data (DRUGyyQq) was merged into a single table that contains both legacy and current case identifiers (*isr* and *primaryid*). Only one case (the latest one) will found present if we have reports for the case in both the legacy and the current data. With the purpose of portability for drug safety studies and pharmacovigilance, some original fields have been suppressed, but can easily be retrieved when joining this resource with the original FDA data, via the case identifiers.

### Missing value imputation

We perform single missing value imputation on the four fields used in the case de-duplication. We define that at least one version of the case must have all four 'key' demographic data fields (event date, age, sex, reporter country) fully populated. The maximum demographic key values from the fully populated case versions are used to impute single missing values in other versions of the same case. Please note that we only perform single value imputation, in the event that more than one of the four key data fields are missing, no imputation is applied to it. The imputation is performed prior to the case version de-duplication step which uses those four fields.

### Case de-duplication

In LAERS/FAERS, cases may have multiple versions, in addition to the initial case version, one or more follow-up case versions may exist. Additionally, a case may exist in the legacy LAERS data set and/or in the newer FAERS data set. The case de-duplication logic therefore takes into account the multiple case versions and differences between the two data sets. Specifically, it manages the different unique row keys (*isr* versus *primaryid*) and different reporter country codes used. For this dataset our de-duplication logic first extracts the latest (most recent) case version from all the available cases (across legacy and current data) based on the case id, case initial/follow-up code ('I' or 'F'), the case event date, age, sex, reporter country, a concatenated alphabetic ordered list of case version drug names, and a concatenated alphabetic list of case version reaction preferred terms (outcomes). In case all of these fields are the same, then the most recent case version is determined by data set (LAERS/FAERS), descending unique case key (*isr*/*primaryid*) and filename (which include the year and quarter). We the keep the most current case version and remove all others. If a case exists in LAERS and FAERS data then the most recent FAERS (current data) case version is kept.

We implement a second de-duplication step which further refines the above set of latest case versions. This step eliminates duplicates based the four demographic data fields (event date, age, sex and reporter country) regardless of assigned case number. This step is intended to eliminate duplicates in the scenario where a duplicate case version (based on these four demographic fields) was not linked by the FDA processing logic to the original case version(s).

Probabilistic identification of duplicate cases, which account for differences in missing values or inconsistent spelling, has not been performed but is an area for potential future exploration. A total of 4,928,413 unique FAERS/LAERS cases are left after de-duplication and missing value imputation are performed.

### Drug mappings

In order to provide a standardized resource for the community, we successfully used the OHDSI Vocabulary version 5 to map LAERS/FAERS drug names into RxNorm standard code ingredients and clinical drug forms (for multi-ingredient drugs). We mapped all unique case drug names to RxNorm CUIs and OHDSI standard vocabulary concept identifier. In the process we included non-standard and standard codes in order to identify brand names and ingredients. Subsequently we mapped the non-standard codes back to standard codes (via the vocabulary). Note that all drug roles, including concomitant drugs have been mapped.

The drug mapping process is outlined in the following steps:

1. Using regular expressions, we mapped drug string names to the OHDSI standard vocabulary concepts.
2. In addition FAERS data includes a separate field with some specific active ingredient drug names which we also mapped.
3. New Drug Application (NDA) drug string names were mapped, linking the NDA number to the FDA orange book of NDA ingredients.
4. For the remaining unmapped drug names, a manual mapping process using OHDSI Usagi was performed.

With the steps outlined, we managed to achieve drug name coverage of 93%, including concomitant medications. We observed that the remaining 7% of drug names are a combination of drugs not found in RxNorm (either international products or non-prescription products), drug name spelling errors, non-specific drug names. Our drug name mappings cover over 95% of the total number of cases found in LAERS/FAERS. The first three steps are automated in our source code. We are left with 4,245 unique drugs after our mapping step is performed.

### Indication and reaction mappings

The source LAERS/FAERS data contains all indication and reaction information in preferred MedDRA terms. We used the OHDSI vocabulary to map LAERS/FAERS drug indications and reactions from MedDRA preferred terms to SNOMED-CT standard codes with two steps:

1. A simplified mapping table between MedDRA and SNOMED-CT was created by leveraging the existing OHDSI Vocabulary tables.
2. Using this mapping table, we mapped between preferred terms to SNOMED-CT concepts. We were able to map 64% of indications and 80% of reactions to SNOMED-CT.

All outcomes from the REACyyQq source files are mapped to OHDSI standard concept identifiers and SNOMED-CT concepts. However, not all indications from the INDIyyQq source files are mapped as we only mapped the indications for the latest version of each case (which is what was used for the statistical calculations) and not every indication in the source INDIyyQq files. However, all source data files do include the indications for all case versions, including the duplicate case versions. We are left with 17,671 unique reactions mapped and 14,062 unique indications mapped.

### Generation of drug-outcome pairs

In order to calculate the statistical associations between drugs and adverse events, we first constructed all drug-outcome pairs found in the merged data. As each case is associated with at least one drug, either as primary suspect (PS), secondary suspect (SS), concomitant (C), or interacting (I), the drug-event pairs get constructed by combining each drug found in each case and the associated outcome. A total of 60,666,994 drug-event pairs have been generated from the data.

### Contingency tables and statistics calculation

Using the merged data, we constructed two-by-two contingency tables to produce all our statistical calculations provided in the *drug_outcome* tables described in Data Record 2 section. The contingency tables are generated as show in Table 2.

We include in our resource two pre-computed measures of disproportionality for signal detection in spontaneous reporting systems for adverse drug reactions, as outlined by van Puijenbroek *et al.* ^32^.

For the Proportional Reporting Ratio (PPR) calculation we used:

(1)PPR=a/(a+b)c/(c+d)

In order to calculate the 95% confidence interval we used^33^:

(2)95%CI=eln(PRR)±1.96(1a−1a+b+1c+1c+d)

In terms of the Reporting Odds Ratio (ROR) calculations we used^34^:

(3)ROR=(a/c)(b/d)

And for the 95% confidence interval we have:

(4)95%CI=eln(ROR)±1.96(1a+1b+1c+1d)

We verified the PRR and ROR statistic calculation using the example calculations found in Gavali *et al.* ^35^. Table 3 shows an example of these calculations in our dataset using the drug ingredient *Etanercept* and the outcome: *Injection site pain*.

Given (1), we have a PPR of 21.11113 and a ROR of 22.1693. The PPR upper and lower limits of the 95% confidence interval are 21.35586 and 20.8692 respectively. For the ROR 95% confidence interval upper and lower limits we have 22.43718 and 21.90461.

### Code availability

All code used to generate the dataset is available on a public github repository (https://github.com/ltscomputingllc/faersdbstats). The code is freely available under the terms of the Apache License (http://www.apache.org/licenses/). This code was developed and tested using: OHDSI standard vocabulary (http://www.ohdsi.org/web/athena/) version v5.0 08-JUN-15, which includes: RxNorm version 20150504, SNOMED-CT release INT 20150131, and MedDRA version18.0. For all the database operations, PostgreSQL 9.3 was used. The manual drug name mappings step was performed using OHDSI Usagi (https://github.com/OHDSI/usagi). All presets used to generate this dataset are the defaults found on the github repository for the code.

## Data Records

The dataset is publicly available online at Dryad (Data Citation 1) as zip file which includes eleven tab delimited text files and a README.txt file. The filenames and field specifications are found in the README.txt file, as well as the loading instructions. As a summary, four of the eleven files provide the aggregated, de-duplicated and mapped drug, outcome, and indication data derived from LAERS and FAERS (detailed on Data Record 1). In another four files contain calculated the drug-outcome contingency table, all counts for drug-outcomes, and the PPR and ROR of the drug-outcome pairs with respect to the complete data source, and a drilldown table of drug/outcome information (detailed on Data Record 2). Additionally, we include two files that contain the OHDSI vocabulary and concept list used to map our resource. All of these files are tab delimited and can be loaded on any relational database management system; instructions on how to load them on PostgreSQL and MySQL are included in the README.txt file. We also provide a github repository where the code to re-generate this dataset can be downloaded.

### Data record 1—Aggregated and clean source case report data

**Figure 2:**
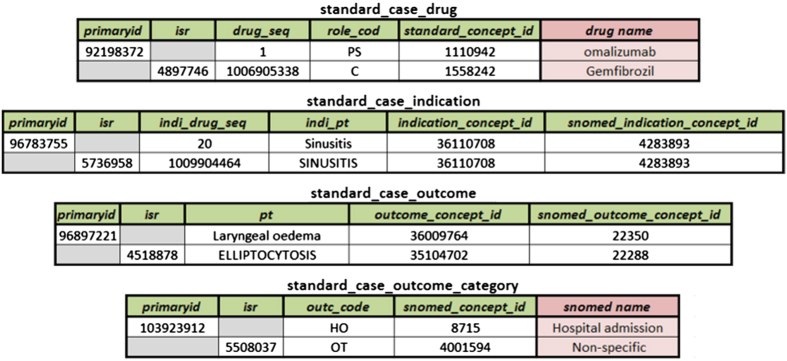
List of tables and sample data for the clean aggregation of the FDA LAERS and FAERS data. Note that the columns in light red are added for presentation clarity and are not included as-is in the actual dataset. The human readable information can be accessed via a join on the respective concept ids.

All aggregated data for the mapped 93% of drugs, with duplicate cases removed, are made available in the four files shown on Fig. 2. Each line corresponds to a particular case report. All cases have drug, indication, and outcome encoded using the OHDSI vocabulary unique concept identifiers, the vocabulary tables needed to map them are found in Data record 3. In the following figures for each table we include all original common FAER/LAERS fields, and the only additions by AEOLUS include the _concept_id fields which include the OHDSI concept identifier mappings and also the corresponding OHDSI concept identifiers for SNOMED concepts.

Note that we maintained both LAERS and FAERS case identifiers intact, *isr* and *primaryid* respectively. This will facilitate joins between this resource and the original FDA data for additional exploratory analysis. When Fig. 2 shows the case identifier field greyed out, this represents that the case number is not found in either LAERS or FAERS.

The standard_case_drug table includes the aggregated and mapped information found in the DRUGyyQq files from LAERS and FAERS. The field drug_seq indicates the drug sequence and role_cod indicates which role the drug played in the case (primary suspect, concomitant, etc.). We aggregated and mapped all REACyyQq files in the resulting table named: standard_case_outcome, for which pt indicates the original textural name of the case outcome. We combined the SNOMED-CT outcome concept identifier, mapped from the original outcome text (field named outc_code), with the OUTyyQq files and generated standard_case_outcome_category. Lastly we provide mapped the indication preferred terms from the INDIyyQq files into OHDSI standard vocabulary concept identifiers and SNOMEDCT concept identifiers and produced standard_case_indication, the original textural indication name is found in the indi_pt field.

### Data record 2—Summary and statistical data for drug-outcome pairs

**Figure 3:**
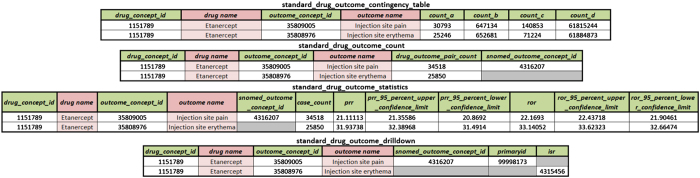
List of files containing drilldown, contingency tables, counts and statistics generated from the aggregate data. Note that the columns in light red are added for presentation clarity and are not included as-is in the actual dataset. The human readable information can be accessed via a join on the respective concept ids.

For our dataset to be instantly useful to the drug safety/PhV community, we calculated contingency tables and summary statistics of the results drug-outcome pairs, the resulting four files are shown in Fig. 3.

Using the aggregated and cleaned source data, we calculated 2×2 contingency tables (in fields count_a, count_b, count_c and count_d) for all drug-outcome combinations found in the data, which are found on the standard_drug_outcome_contingency_table file. The total counts for all drug-outcomes is given in the field drug_outcome_pair_count, which is found in standard_drug_outcome_count. In standard_drug_outcome_drilldown we present the mapped drug/outcome pairs found in all cases. Lastly, we calculated for all drug-outcome pairs the PPR and ROR with their 95% confidence interval (upper and lower values) in the standard_drug_outcome_statistics file. When Fig. 3 shows the case identifier field greyed out, this represents that the case number is not found in either LAERS or FAERS. The outcomes that cannot be mapped to SNOMEDCT concept identifiers are also left in gray.

## Technical Validation

In order to verify that our generation process was successful, we verified that the FDA source LAERS/FAERS data matched in terms of record counts to the pre-processed version we created. We selected a random sample of ten unique case files that included multiple case versions (from both LAERS and FAERS) using the FDA’s FAERS application programming interface (API). For each of the random case files we performed the following checks:

1. If the same (latest) case version was found via the OpenFDA API, this would indicate that the initial versus follow-up case de-duplication process was performed successfully.
2. Verified that a comparable list of drugs was retrieved by the OpenFDA API for each case tested.
3. Verified that a comparable list of outcomes/reactions was retrieved by the OpenFDA API for each case tested.

In order to validate the drug mappings we manually reviewed a small sample by empirically verifying that the RxNorm CUI and name matched with the proper OHDSI concept and source concept fields. The same process was performed to verify small sample of the brand name to ingredient or clinical drug form mappings.

In terms of validating the drug/outcome counts, other than using the verification of the original data versus the curated set, it is quite hard as the counts on our dataset are dependent of the drug mapping algorithm. The choice of algorithm, and level of rigor, will determine how the mappings are performed greatly impacting the number of drug-outcome pairs. This validation is also highly dependent on de-duplication strategies that would yield less or more cases, thus increasing the drug-outcome pair counts. While there is no established way of validating this section, we strongly believe that having performed due diligence when verifying the data consistency and quality of the mappings, will be enough to have proper drug-outcome pairs.

## Usage Notes

This resource aims to alleviate curation and mapping efforts done by independent researchers to produce reliable FAERS data. AEOLUS differs to the Sentinel efforts in the sense that these are about mining the EHR data as a complementary source to the data from submitted adverse event reports. In turn, AEOLUS makes the FARES reports available in a clean form to anyone and would make the analysis of EHR data in conjunction with FAERS data accessible to all researchers. In the following use-cases we present scenarios where researchers have used self-curated FAERS data in studies related to drug safety and pharmacovigilance. The use cases provide concrete examples of the kind of studies that would benefit from a publically available, clean copy of FAERS.

### Discovery of adverse events using clinical notes

Wang and Jung *et al.* ^36^ demonstrated a method for systematic discovery of adverse drug events from clinical notes, and have shown that post-marketing surveillance for ADEs using electronic medical records is possible. Their method uses the contents of clinical notes, along with prior knowledge of drug usages and known ADEs, as inputs to discriminative classifier which outputs the probability that a given drug-disorder pair represents a potential ADE association. The authors validated their approach based on the degree of support drug-outcome pairs had from FAERS and MEDLINE.

Tatonetti *et al.* ^37^ developed a comprehensive database of drug effects and drug-drug interaction side effects (known as OFFSIDES and TWOSIDES, respectively) based on mining the FAERS data. The database was used to calculate drug-outcomes counts in order to form PRR values. Since calculating case counts and PRR values are generally computationally intensive, providing these values in the summary tables can be useful to independent researchers. Additionally, by providing drug mappings to RxNorm and grouping drugs via ATC classes, associations between drug classes and adverse effects can be easily computed. Similarly, by mapping outcomes to SNOMED-CT identifiers, adverse events can also be grouped in order to find outcome-drug associations, or even outcome-drug class associations. For example, hypertension and hypotension can be grouped to find associations to drugs that affect blood pressure generally.

### Integrating evidence within an interactive product label

Structured product labels (SPL) provide an electronic representation of the FDA-approval and manufacturer-provided information that is communicated to health care providers as education about the composition and effects of pharmaceuticals. The product label represents a summary of evidence compiled from multiple sources, including clinical trials, observational studies, and spontaneous reports. A key challenge to interpreting the product label is that the evidence that was used to generate the summary is not directly accessible, and as such, some of the context is missing. A product label may list that an adverse event has been observed in post-marketing experience, but does not provide the number of cases observed or incidence rate estimates that would allow a reader to understand the relative frequency of the event. OHDSI has compiled evidence from published literature, product labels, observational data, and spontaneous reporting into one common harmonized evidence base^38^. This evidence can then be exposed through an interactive web-based representation of the structured product label, such that users who want to learn more about a purported safety effect can drilldown from the simple mention in the label to the totality of the evidence that is known about the effect. Data from FAERS offers insights into how often the event has been reported overall, the disproportionality by which it is co-reported with the drug of interest, the seriousness of the cases, and the reported outcomes associated with the events. Together with real-world evidence from observational data and summaries from literature, the tool provides the necessary context to interpret the scope and severity of potential safety issues when informing medical decision-making.

### Identifying drug-outcome associations to empirically calibrate observational research

Observational research is plagued by the combination of random and systematic error that can persist in analyses and limit the appropriate interpretation of study findings. Bias in epidemiologic research has resulted in observational studies generating treatment effect estimates that have been subsequently demonstrated through randomized clinical trial to be entirely incorrect. Recent advances in observational analysis methodology have identified mechanisms for providing context around and potentially overcoming some of the issues associated with systematic error. Schuemie *et al.* ^39^. proposed a novel method for empirical calibration of *P*-values in observational studies that relies on selection of drug-outcome negative controls (drugs known not to be associated with an events). Since negative controls can be assumed to have no effect, methods estimating the effect should yield estimates close to relative risk=1. The observed deviation from the null effect across a sample of negative controls can be used to empirically derive a null distribution, which can subsequently be used to calibrate *P*-values. Ryan *et al.* ^38^ highlighted a process for identifying negative controls to be used for empirical calibration. Integral to that process of ensuring that a drug is not associated with an outcome is evaluating the spontaneous reporting database to confirm that a disproportionate number of adverse events have not been previously reported. FAERS can be used to produce candidate negative control drug-outcome pairs, which can be cross-checked with other evidence sources and adjudicated by clinical expert review in order to provide evidence about observational research performance and improve the integrity of observational study results.

### Prioritization of potential drug-drug interactions

Methods to identify drug interactions make statistically plausible drug-drug interaction predictions, which range from a few hundred to thousands. For example, Iyer *et al.* ^40^ demonstrated the feasibility of identifying drug-drug interactions and estimating the rate of adverse events among patients on drug combinations, directly from clinical data^40^. They identified 5,983 putative drug-drug interactions, and published a database of adverse event rates among patients on drug combinations based on an EHR corpus. In order to prioritize which interactions are most likely to be true and should be further investigated, Banda *et al.* ^41^ developed a proof-of-concept framework to prioritize these 5,983 drug-drug interactions. This framework requires gathering MEDLINE and FAERS data to rank potential associations based on overlap with these sources. In this work the authors, performed their own independent curation of FAERS to identify potential associations—a task that would have been significantly faster if a resource such as the one we present already existed.

### Flexibility of the resource

One major advantage of this resource is the mapping of the outcomes and indications to SNOMED-CT, which will allow researchers to link out to other ontologies—such as International Classification of Diseases (ICD) codes—using mappings from the Unified Medical Language System (UMLS). Such mapping to SNOMED-CT was never made publicly available before.

By making all the drug mappings to RxNorm and the standard OHDSI vocabulary concept identifiers, we are now able to group drugs via ATC classes, VA Class, and NDFRT. This mapping also makes possible the linkage to other existing drug safety resources like LAERTES^42^ and drug-drug interaction datasets such as^43^ and^44^.

Finally, while similar efforts to ours have been done in the past^45^, they do not provide source code and the data becomes out of date rapidly. Another major advantage of our resource is that we provide all source code for researchers to periodically refresh the data quarterly as the FDA releases new FAERS data. By releasing all documentation and code we are enabling all researchers to update the dataset when they need to, rather than having them wait for our group to release a new version (when funding and time permits).

## Additional Information

**How to cite this article:** Banda, J. M. *et al.* A curated and standardized adverse drug event resource to accelerate drug safety research. *Sci. Data* 3:160026 doi: 10.1038/sdata.2016.26 (2016).

## Supplementary Material



## Figures and Tables

**Table 1:** FAERS/LAERS structure of source data

**Filename**	**Description**
DEMOyyQq	Contains patient demographic and administrative information, each row represent an individual event report
DRUGyyQq	Contains drug information for all medications reported for the event report (1 or more rows per report)
INDIyyQq	Contains all MedDRA terms for the indications of use for the reported drugs (0 or more per drug per event)
OUTCyyQq	Contains patient outcomes for the event report (0 or more rows per report)
REACyyQq	Contains all MedDRA terms related to the adverse event report (1 or more rows per report)
RPSRyyQq	Contains the source of the event report (0 or more rows per report)
THERyyQq	Contains drug therapy start dates and end dates for the reported drugs (0 or more rows per report)
Note that yyQq represents year and quarter in each file.	

**Table 2:** Two-by-two contingency table.

	**Reports with the suspected ADR**	**Reports without the suspected ADR**
Reports with the suspected drug	a	b
All other reports	c	d

**Table 3:** Two-by-two contingency table for example ADR.

	**Reports with** ***Injection site pain***	**Reports without** ***Injection site pain***
Reports with *Etanercept*	30,793	647,134
All other reports	140,853	61,815,244
